# Clinical Significance of Pre-to-Postoperative Dynamics of Aspartate Transaminase/Alanine Transaminase Ratio in Predicting the Prognosis of Renal Cell Carcinoma after Surgical Treatment

**DOI:** 10.1155/2020/8887605

**Published:** 2020-07-04

**Authors:** Minyong Kang, Seung Jea Shin, Hyun Hwan Sung, Hwang Gyun Jeon, Byong Chang Jeong, Seong Soo Jeon, Hyun Moo Lee, Seong Il Seo

**Affiliations:** ^1^Department of Urology, Samsung Medical Center, Sungkyunkwan University College of Medicine, Seoul, Republic of Korea; ^2^Department of Health Sciences and Technology, SAIHST, Sungkyunkwan University, Seoul, Republic of Korea; ^3^Blue Urology Clinic, Seoul, Republic of Korea

## Abstract

**Background:**

This study is aimed at examining the prognostic role of pre-to-postoperative dynamics of De Ritis ratio (aspartate aminotransaminase (AST)/alanine aminotransaminase (ALT)) in patients with nonmetastatic renal cell carcinoma (RCC) following radical nephrectomy.

**Methods:**

We retrospectively reviewed the records of 670 patients who underwent radical nephrectomy for nonmetastatic RCC between 1996 and 2012 at our institution. The cutoff points for preoperative (=1.0) and postoperative AST/ALT ratios (=1.12) were assigned based on the median values. We categorized patients into four groups according to the dynamics of AST/ALT ratios: group 1 (lower (≤1.0) ⟶ lower (≤1.12)), group 2 (lower (≤1.0) ⟶ higher (>1.12)), group 3 (higher (>1.0) ⟶ lower (≤1.12)), and group 4 (higher (>1.0) → higher (>1.12)).

**Results:**

When grouped by a preoperative AST/ALT ratio alone, the groups were not statistically different in cancer-specific survival (CSS) or overall survival (OS). In contrast, in Kaplan-Meier analysis, CSS (*P* = 0.0296) and OS (*P* = 0.0324) were both significantly shorter with an increased postoperative AST/ALT ratio. According to the pre-to-postoperative dynamics of the AST/ALT ratio, group 2 (lower (≤1.0) ⟶ higher (>1.12)) had a significantly lower CSS (*P* = 0.0028) and OS (*P* = 0.0194) than the other groups. On multivariate Cox regression analysis, the pre-to-postoperative dynamics of the AST/ALT ratio were a significant prognostic factor for CSS (hazard ratio, HR = 3.45) and OS (HR = 2.18).

**Conclusion:**

This study is the first to suggest that the dynamics of the pre-to-postoperative De Ritis ratio represent an independent prognostic factor for RCC patients following nephrectomy.

## 1. Introduction

Among patients with nonmetastatic renal cell carcinoma (RCC), up to 20 to 30% suffer recurrence after curative surgery [1]. Postoperative surveillance is standard, and no adjuvant treatment for patients at a high risk of recurrence has been established yet [2, 3]. Thus, it is important to identify individuals at a high risk of recurrence who can benefit from close surveillance and early detection in order to improve the survival rate of RCC patients who undergo nephrectomy. A number of studies have reported various prognostic factors that can stratify high-risk patients [4]. Although a risk score, prognostic models, and nomograms using tumor characteristics and genomic markers have been studied [5–7], there are no optimal prognostic markers to identify the patients with a high risk of tumor recurrence yet.

Recently, researchers have been focusing on the prognostic value of a preoperative aspartate aminotransaminase (AST)/alanine aminotransaminase (ALT) (De Ritis) ratio in patients with nonmetastatic RCC who underwent radical nephrectomy [8, 9]. Bezan and colleagues reported that the preoperatively measured AST/ALT ratio was an independent prognostic factor for metastasis-free survival and OS in patients with nonmetastatic RCC. This ratio improved the predictive accuracy when added to the Harrell c-index, a well-established prognosis score [8]. Another study analyzed the association between AST/ALT ratio and postoperative outcome of RCC after curative nephrectomy using propensity score matching. In this study, the preoperative AST/ALT ratio was a significant prognostic factor for cancer-specific survival (CSS) as well as overall survival (OS) [9].

Both previous studies, however, focused on preoperative AST/ALT ratio alone, and the prognostic value of postoperative and pre-to-postoperative dynamics of the AST/ALT ratio remains unexplored. We hypothesized that dynamic features of the pre- and postoperative AST/ALT ratio can have a prognostic value, given the prognostic effect of the preoperative AST/ALT ratio. Here, we evaluated the potential prognostic significance of pre-to-postoperative dynamics of the AST/ALT ratio on survival in patients with RCC who underwent radical nephrectomy.

## 2. Materials and Methods

### 2.1. Study Population

We collected and reviewed the clinicopathological data of 785 patients with RCC who underwent radical nephrectomy between October 1996 and December 2012 at Samsung Medical Center. Among these, 115 patients were excluded from our analysis for the following reasons: synchronous metastasis (*n* = 63), insufficient follow-up period less than 6 months after surgery (*n* = 10), and no AST and/or ALT values before and/or 6 months after surgery (*n* = 42). A total of 670 patients with RCC treated by radical nephrectomy were included in the final analysis.

### 2.2. Study Design

Clinical data on age at surgery, sex, height, weight, and body mass index (BMI) were recorded at admission for operation. An abdomen and pelvis computed tomography (CT) scan and a chest CT scan were performed before surgery in all patients to assess the status of regional lymph nodes and the presence of distant metastases. Aminotransaminase (AST/ALT) levels were measured within 1 month prior to surgery and 6 months after surgery. The median preoperative AST/ALT ratio was 1.00 and the postoperative AST/ALT ratio was 1.12, respectively. The cutoff points for preoperative and postoperative AST/ALT ratios were assigned based on the median values of each. To evaluate the prognostic impact of the dynamics of the pre-to-postoperative AST/ALT ratio on survival outcomes, we classified patients into four different groups according to the dynamics of AST/ALT ratios between preoperative and postoperative status as follows: group 1 (lower (≤1.0) to lower (≤1.12) level), group 2 (lower (≤1.0) to higher (>1.12)), group 3 (higher (>1.0) to lower (≤1.12)), and group 4 (higher (>1.0) to higher (>1.12)).

Tumor size was defined as the longest dimension of the tumor in preoperative CT images. The assessment of pathology was performed by experienced pathologists at our center. Tumor stage was classified by the American Joint Committee on Cancer/Union for International Cancer Control TNM system [10]. The Fuhrman grading system was used to measure nuclear grade [11]. Surgical procedures included one of three types: open surgery, hand-assisted laparoscopic surgery, and pure laparoscopic surgery.

The primary endpoint was overall survival following radical nephrectomy. The secondary endpoint was cancer-specific survival after surgery. All patients were routinely followed up according to the standard protocol in our institution. We assessed medical history, physical examinations, laboratory tests, and imaging screening, such as an abdomen and pelvis CT scan and/or a chest CT scan, at intervals of 3 to 6 months during the first 2 years, and yearly thereafter. Death information for all patients was collected based on the vital record data of our institution, which incorporated data from the Korean National Statistical Office.

### 2.3. Statistical Analyses

Descriptive analysis of demographic and clinicopathological variables was performed. Data are shown as median values with interquartile range (IQR) or by the frequency of relevant events. For group comparisons according to dynamics of the AST/ALT ratio, we used a chi-square test for categorical variables and the Mann-Whitney *U* test or Kruskal-Wallis test for continuous variables.

We estimated and compared survival outcomes between groups using Kaplan-Meier survival analysis with a log-rank test. Multivariate Cox proportional hazards regression analysis was performed to identify the predictors of survival outcomes after adjusting for potential confounding factors.

The results are described as hazard ratios (HR) with 95% confidence intervals (CI). *P* values < 0.05 or those that did not overlap the zero point with their 95% CI were considered statistically significant. Statistical analyses were conducted using SPSS Statistics version 23.0 (SPSS, Inc., Chicago, IL, USA) and GraphPad Prism software version 8.0 (GraphPad Software Inc., San Diego, CA, USA).

## 3. Results

Table 1 shows the baseline characteristics of 670 patients with RCC in this study. The median age of patients at surgery was 55 years (IQR = 48–64). The histology of RCC was clear cell type in 573 patients (85.5%) and nonclear cell type in 93 patients (13.9%). Approximately 77% of patients were with a localized stage (pT1–2) (*n* = 520), and patients with a locally advanced stage were 22.4% (*n* = 150). Majority of patients had a high-grade (nuclear grade 3–4) tumor (68.8%), and only 29.6% of patients had a low-grade (nuclear grade 1–2) tumor. Median follow-up duration was 59 months (IQR = 41–81). A total of 108 patients (16.1%) died during follow-up, of which 78 (11.6%) died from RCC.

Based on pre-to-postoperative dynamics of the AST/ALT ratio, the number of patients in group 1 (lower (≤1.0) ⟶ lower (≤1.12)), group 2 (lower (≤1.0) ⟶ higher (>1.12)), group 3 (higher (>1.0) ⟶ lower (≤1.12)), and group 4 (higher (>1.0) ⟶ higher (>1.12)) was 228 (34.0%), 89 (13.3%), 114 (17.0%), and 239 (35.7%), respectively. Table S1 shows the comparison of clinicopathological characteristics of patients according to pre-to-postoperative dynamics of the AST/ALT ratio. Notably, we found that there were no significant differences in tumor size, T stage, and Fuhrman grade between the four groups.

**Figure 1:**
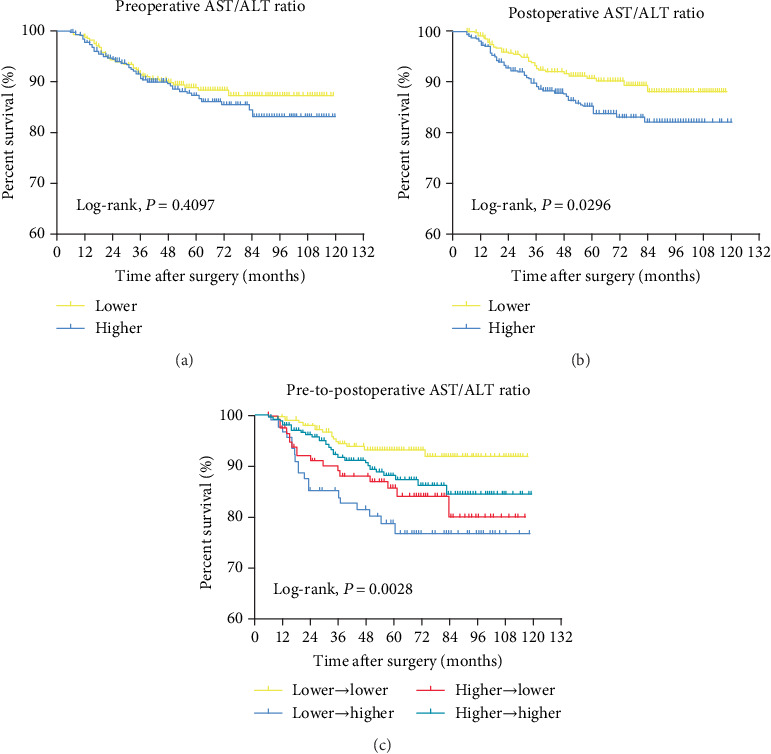
Kaplan-Meier analysis for estimating CSS according to various types of the De Ritis ratio.

**Figure 2:**
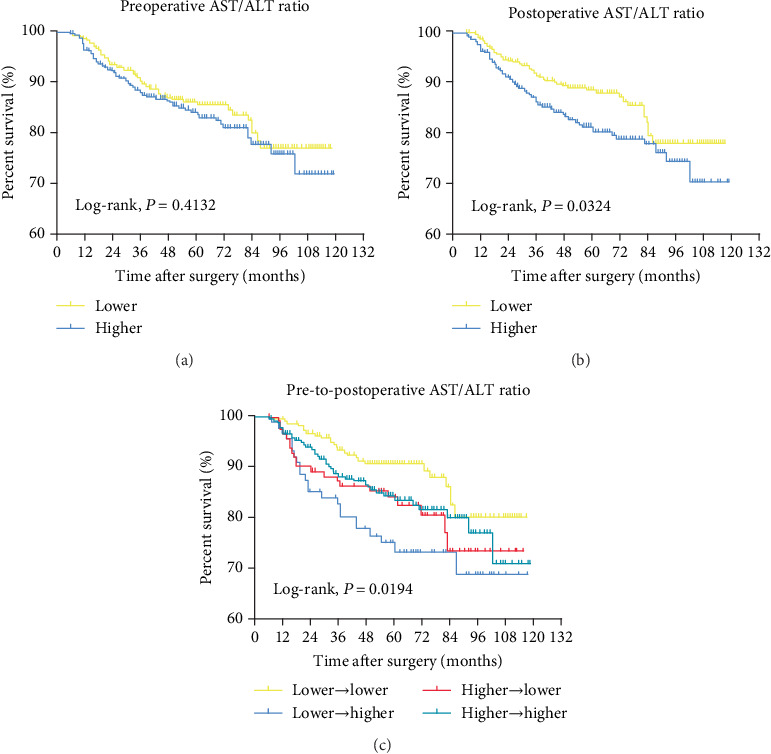
Kaplan-Meier analysis for estimating OS according to various types of the De Ritis ratio.

Figures 1 and 2 depict Kaplan-Meier analyses for estimating CSS and OS, respectively, according to preoperative, postoperative, and pre-to-postoperative dynamics of the AST/ALT ratio. While there were no statistical differences in either CSS or OS according to the preoperative AST/ALT ratio, patients with a higher postoperative AST/ALT ratio had worse CSS and OS compared to those with a lower postoperative AST/ALT ratio. Of note, among four groups by pre-to-postoperative dynamics of the AST/ALT ratio, patients in group 1 (lower (≤1.0) ⟶ lower (≤1.12)) showed significantly better CSS and OS outcomes compared to other three groups. However, patients in group 2 (lower (≤1.0) ⟶ higher (>1.12)) had poorer CSS and OS outcomes than other three groups.

More importantly, multivariate analysis revealed that the postoperative AST/ALT ratio (hazard ratio, HR: 1.88, 95% CI: 1.18–2.99) and pre-to-postoperative dynamics of the AST/ALT ratio (HR: 3.45, 95% CI: 1.73–6.88) remained as significant prognostic factors for CSS, in addition to tumor size (HR: 4.23, 95% CI: 2.66–6.72) and pT stage (HR: 2.45, 95% CI: 1.53–3.94) (Table 2). We also identified pre-to-postoperative dynamics of the AST/ALT ratio (HR: 2.18, 95% CI: 1.23–3.89) as an independent predictor of OS, as well as tumor size (HR: 2.91, 95% CI: 1.93–4.40), pT stage (HR: 2.20, 95% CI: 1.47–3.28), and age at surgery (HR: 2.03, 95% CI: 1.36–3.02), as shown in Table 3.

## 4. Discussion

A number of studies have been conducted on the prognostic factors that predict high-risk RCC patients. Leibovich et al. developed a prognosis score that stratifies the risk of progression to metastasis after radical nephrectomy [4]. Risk score, prognostic modeling, and nomograms were also evaluated for predicting survival in patients with nonmetastatic RCC following nephrectomy [5, 6]. In addition, several prognostic biomarkers related to survival in RCC are being proposed in line with the development of genetics, including VHL, CRP, ESR, PD-L1/B7-H1, Ki-67, survivin, c-Met, and IMP3 [7]. However, most of these biomarkers are of tissue origin, expensive to measure, and have a low reproducibility.

AST is widely synthesized in different types of tissues, whereas synthesis of ALT is thought to be more liver specific [12]. Therefore, pathological processes leading to a higher proliferative state, tissue damage, or increased tumor cell turnover tend to increase circulating AST levels, but not ALT, at least to the same extent. These characteristics make the AST/ALT ratio an attractive biomarker in cancer patients. Hence, the AST/ALT ratio has received attention as a simple, inexpensive, and easily applicable serum biomarker.

Recently published articles have addressed the prognostic significance of the preoperative AST/ALT (De Ritis) ratio on prognosis in nonmetastatic RCC after surgery [8, 9, 13] and metastatic RCC with tyrosine kinase inhibitor therapy [14]. In patients with localized RCC, Bezan et al. revealed that an elevated preoperative AST/ALT ratio (>1.26) was independently associated with poor OS (HR = 1.76) and metastasis-free survival (HR = 1.61). When the AST/ALT ratio was added to the prognosis score, it significantly improved the predictive accuracy [8]. Lee et al. also showed a higher AST/ALT ratio (>1.5) as a valuable prognostic marker of CSS (HR = 1.97), OS (HR = 1.56), and progression-free survival (HR = 1.37) in patients with clear cell RCC histology [9]. Hamilton et al. proposed novel combination of preoperative tumor morphology (RENAL score) and AST/ALT ratio to be associated with worsened OS in localized RCC. In the COX model for OS, the increased AST/ALT ratio (>1.5) was significantly associated with worsened OS (HR = 2.25) [13]. In metastatic RCC patients treated by targeted therapy, our research team evaluated the prognostic impact of the pretreatment AST/ALT ratio and reported that patients with a higher pretreatment AST/ALT ratio (>1.12) had worse CSS and OS than those with a lower AST/ALT ratio [14]. On the contrary, Canat et al. demonstrated that the increased preoperative AST/ALT ratio (>1.5) did not represent an independent prognostic factor with respect to OS and CSS in patients with localized RCC, although this ratio had a significant association with renal vein invasion, renal capsule infiltration, and renal pelvis involvement in localized RCC [15].

However, underlying liver disease or other malignancies could be confounding factors that influence the metabolism of both AST and ALT [12, 16, 17]. Moreover, serum activity of aminotransferase may be affected by a variety of factors including BMI, triglyceride levels, cholesterol levels, and alcohol consumption [18–20]. For these reasons, aminotransferase measured just once before treatment might be nonspecific and affected by numerous factors other than RCC status. In contrast, in terms of pre-to-postoperative dynamics of the AST/ALT ratio, host conditions other than RCC status are almost the same before and after surgery. Hence, pre-to-postoperative dynamics of the AST/ALT ratio might have a little effect on other basement characteristics. However, previous studies have focused on the pretreatment of the AST/ALT ratio, and the prognostic role of pre-to-post-treatment dynamics of the AST/ALT ratio is unknown.

To the best of our knowledge, this study is the first to suggest the prognostic value of the dynamics of the pre-to-postoperative AST/ALT ratio in localized RCC patients following radical nephrectomy. With regard to the preoperative AST/ALT ratio, Bezan et al. suggested that the elevated aerobic glycolysis and pyruvate production in tumor cells, which is the Warburg effect, was the key biological mechanism of the poor survival outcomes in patients with elevated De Ritis ratio [8]. Because there is no visible tumor after surgery in patients with nonmetastatic RCC, the potential mechanism of a postoperative increase of De Ritis ratio can be explained as follows: First, an isolated elevation of AST can occur by a nonhepatic source of AST [21]. This event artefactually occurs by the release of AST from blood cells due to sample hemolysis [21]. Second, isolated elevation of the AST value often occurred by the damage of the nonliver cells, particularly muscle cells containing mitochondria [21, 22]. Since the major surgery could significantly affect to the general condition of the patients, some patients may experience the muscle injury and the loss of muscle volume after surgery. Finally, serum AST and ALT levels have been used as a systemic inflammatory marker in various diseases [23]. Considering the presence of inflammatory response is associated with survival outcomes in patients with various malignancies, major surgery could influence the host condition and systemic inflammatory status was changed, including the elevated AST/ALT ratio after surgery.

In our results, patients in group 2 (lower (≤1.0) ⟶ higher (>1.12)) showed significantly worse oncological outcomes compared to the other groups. Considering there were no significant differences in tumor size, pT stage, and Fuhrman grade between the four groups, these results indicate that the pre-to-postoperative dynamics of the AST/ALT ratio can be a valuable prognostic marker in patients with RCC who were treated by surgery. Interestingly, patients with an increased AST/ALT ratio (lower ⟶ higher) had poorer prognosis than those with a persistently elevated AST/ALT ratio (higher ⟶ higher). Based on these findings, we assume that an increasing tendency of the AST/ALT ratio from the preoperative basement, rather than the AST/ALT ratio itself, is more closely related to worse survival outcomes, even in those with a higher postoperative AST/ALT ratio.

We acknowledged that our study has several limitations. First, our findings should be interpreted in the context of the retrospective design, which might possess an inherent structural bias. However, we performed consecutive patient sampling to reduce selection bias and tried to obtain patients' information as completely as possible. Second, our study only included patients that underwent radical nephrectomy, and therefore, a distinction to those who are treated by nephron-sparing surgery should be examined in a further study. Third, we did not adjust the analyses for potentially confounding factors such as liver diseases or other metabolic conditions that may significantly impact on the levels of aminotransferase and therefore on the AST/ALT ratio. Fourth, we did not conduct a subanalysis based on histological types of RCC. Fifth, patients with disease progression underwent different kinds of salvage therapies according to their treating clinicians and timing, which may have influenced survival outcomes. Additionally, our dataset lacks additional information known to be associated with poor prognosis at presentation such as performance status, the comorbidity status, and other clinical variables such as calcium, hemoglobin, platelet, and neutrophil counts. Finally, our study was based on only Korean patients and should be validated before the results can be applied to other racial groups.

## 5. Conclusion

In conclusion, our data revealed that the postoperative AST/ALT ratio was identified as a better prognostic factor for survival than the preoperative AST/ALT ratio in patients who were surgically treated for nonmetastatic RCC. Furthermore, the pre-to-postoperative dynamics of the AST/ALT ratio have prognostic significance for postoperative survival in these patients, particularly in those with lower preoperative to higher postoperative AST/ALT ratio. Our findings suggest the potential use of this simple serum marker, particularly dynamics of pre-to-postoperative status, as a valuable prognostic factor of survival outcomes after radical nephrectomy in patients with nonmetastatic RCC.

## Figures and Tables

**Table 1:** Baseline demographics of patients with renal cell carcinoma following radical nephrectomy.

Clinicopathological variables	
No. of patients	670
Age at surgery	
Median (yr)	55 (48–64)
<60 years	411 (61.3)
≥60 years	259 (38.7)
Sex	
Male	473 (70.6)
Female	197 (29.4)
BMI (kg/m^2^)	
Median	24.5 (22.4–28.6)
<25	370 (55.2)
≥25	300 (44.8)
Preoperative AST/ALT ratio	
Median	1.00 (0.79–1.27)
≤1.00 (lower)	317 (47.3)
>1.00 (higher)	353 (52.7)
Postoperative AST/ALT ratio	
Median	1.12 (0.86–1.36)
≤1.12 (lower)	341 (50.9)
>1.12 (higher)	329 (49.1)
AST/ALT dynamics (pre-to-postoperative)	
Lower → lower	228 (34.0)
Lower → higher	89 (13.3)
Higher → lower	114 (17.0)
Higher → higher	239 (35.7)
Laterality	
Left	322 (48.1)
Right	348 (51.9)
Tumor size (cm)	
Median	5.0 (3.9–6.5)
<7.0	546 (81.5)
≥7.0	124 (18.5)
Histology	
Clear cell	573 (85.5)
Nonclear cell	93 (13.9)
*Missing data*	*4 (0.6)*
pT stage	
pT1–2	520 (77.6)
pT3–4	150 (22.4)
Fuhrman grade	
Low (grade 1–2)	198 (29.6)
High (grade 3–4)	461 (68.8)
*Missing data*	*11 (1.6)*
Survival outcomes	
All-cause death	108 (16.1)
Cancer-specific death	78 (11.6)
Median F/U duration (mon)	59 (41–81)

**Table 2:** Multivariate Cox regression analyses to identify predictors for cancer-specific survival in patients with renal cell carcinoma following radical nephrectomy.

Variables	Univariate			Multivariate		
	Hazard ratio	95% CI	*P* value	Hazard ratio	95% CI	*P* value
Age at surgery						
<60 years	Reference					
≥60 years	1.22	0.77–1.92	0.382			
Sex						
Male	Reference					
Female	0.65	0.38–1.12	0.128			
BMI (kg/m^2^)						
<25	Reference			Reference		
≥25	0.53	0.32–0.85	0.009	0.64	0.39–1.06	0.082
Preoperative AST/ALT ratio						
≤1.00 (lower)	Reference					
>1.00 (higher)	1.21	0.77–1.88	0.411			
Postoperative AST/ALT ratio						
≤1.12 (lower)	Reference			Reference		
>1.12 (higher)	1.64	1.04–2.59	0.031	1.88	1.18–2.99	0.008
AST/ALT dynamics						
Lower → lower	Reference			Reference		
Lower → higher	3.37	1.71–6.64	<0.001	3.45	1.73–6.88	<0.001
Higher → lower	2.34	1.17–4.69	0.016	1.70	0.83–3.46	0.145
Higher → higher	1.81	0.96–3.41	0.064	1.75	0.92–3.31	0.087
Tumor size						
<7 cm	Reference			Reference		
≥7 cm	4.78	3.06–7.48	<0.001	4.23	2.66–6.72	<0.001
Histology						
Clear cell	Reference					
Nonclear cell	0.97	0.49–1.88	0.927			
pT stage						
pT1–2	Reference			Reference		
pT3–4	3.50	2.24–5.46	<0.001	2.45	1.53–3.94	<0.001
Fuhrman grade						
Low	Reference			Reference		
High	2.43	1.31–4.50	0.005	1.72	0.91–3.29	0.096

**Table 3:** Multivariate Cox regression analyses to identify predictors for overall survival in patients with renal cell carcinoma following radical nephrectomy.

Variables	Univariate			Multivariate		
	Hazard ratio	95% CI	*P* value	Hazard ratio	95% CI	*P* value
Age at surgery						
<60 years	Reference			Reference		
≥60 years	2.16	1.48–3.16	<0.001	2.03	1.36–3.02	0.008
Sex						
Male	Reference					
Female	0.63	0.39–1.01	0.052			
BMI (kg/m^2^)						
<25	Reference			Reference		
≥25	0.61	0.41–0.91	0.014	0.68	0.45–1.03	0.068
Preoperative AST/ALT ratio						
≤1.00 (lower)	Reference					
>1.00 (higher)	1.17	0.80–1.71	0.414			
Postoperative AST/ALT ratio						
≤1.12 (lower)	Reference			Reference		
>1.12 (higher)	1.51	1.03–2.21	0.034	1.43	0.96–2.13	0.074
AST/ALT dynamics						
Lower → lower	Reference			Reference		
Lower → higher	2.41	1.36–4.25	0.002	2.18	1.23–3.89	0.008
Higher → lower	1.74	0.97–3.10	0.062	1.19	0.65–2.17	0.571
Higher → higher	1.57	0.95–2.60	0.077	1.24	0.74–2.08	0.417
Tumor size (cm)						
<7	Reference			Reference		
≥7	3.12	2.09–4.62	<0.001	2.91	1.93–4.40	<0.001
Histology						
Clear cell	Reference					
Nonclear cell	1.09	0.63–1.88	0.759			
pT stage						
pT1–2	Reference			Reference		
pT3–4	3.11	2.12–4.56	<0.001	2.20	1.47–3.28	<0.001
Fuhrman grade						
Low	Reference			Reference		
High	1.17	1.10–2.83	0.018	1.29	0.79–2.12	0.303

## Data Availability

Data are available on request.

## Abbreviations

RCC: Renal cell carcinoma
AST: Aspartate aminotransaminase
ALT: Alanine aminotransaminase
CSS: Cancer-specific survival
OS: Overall survival
BMI: Body mass index
CT: Computed tomography
IQR: Interquartile range
HR: Hazard ratios
CI: Confidence intervals.

## Supplementary Materials

Supplementary Materials Table S1: Comparison of clinicopathological characteristics of patients with renal cell carcinoma following radical nephrectomy according to pre-to-postoperative AST/ALT dynamics.
