# Supplementary material for: Clinical Significance of Pre-to-Postoperative Dynamics of Aspartate Transaminase/Alanine Transaminase Ratio in Predicting the Prognosis of Renal Cell Carcinoma after Surgical Treatment
Source: Dis Markers. 2020 Jul 4;2020:8887605. doi: 10.1155/2020/8887605 (PMC7364199; doi:10.1155/2020/8887605)
Supplement: Supplementary Materials — Table S1: Comparison of clinicopathological characteristics of patients with renal cell carcinoma following radical nephrectomy according to pre-to-postoperative AST/ALT dynamics. [file 8887605.f1.docx]

Table S1: Comparison of clinicopathological characteristics of patients with renal cell carcinoma following radical nephrectomy according to pre-to-postoperative AST/ALT dynamics.

| **Variables** | **Group 1**  **(Lower→Lower)** | **Group 2**  **(Lower→Higher)** | **Group 3**  **(Higher→Lower)** | **Group 4**  **(Higher→Lower)** | ***p* value** |
| --- | --- | --- | --- | --- | --- |
| No. of patients (%) | 228 (34.0) | 89 (13.3) | 114 (17.0) | 239 (35.7) |  |
| Age at surgery |  |  |  |  |  |
| < 60 years | 158 (69.3) | 59 (66.3) | 68 (59.6) | 126 (52.7) | 0.002 |
| ≥ 60 years | 70 (30.7) | 30 (33.7) | 46 (40.4) | 113 (47.3) |  |
| Sex |  |  |  |  |  |
| Male | 190 (83.3) | 66 (74.2) | 87 (76.3) | 130 (54.4) | < 0.001 |
| Female | 38 (16.7) | 23 (25.8) | 27 (23.7) | 109 (45.6) |  |
| BMI (kg/m^2^) |  |  |  |  |  |
| < 25 | 86 (37.7) | 41 (46.1) | 73 (64.0) | 179 (71.1) | < 0.001 |
| ≥ 25 | 142 (62.3) | 48 (53.9) | 41 (36.0) | 69 (28.9) |  |
| Laterality |  |  |  |  |  |
| Left | 114 (50.0) | 46 (51.7) | 51 (44.7) | 111 (46.4) | 0.667 |
| Right | 114 (50.0) | 43 (48.3) | 63 (55.3) | 128 (53.6) |  |
| Tumor size (cm) |  |  |  |  |  |
| < 7.0 | 190 (83.3) | 74 (83.1) | 87 (76.3) | 195 (81.6) | 0.440 |
| ≥ 7.0 | 38 (16.7) | 15 (16.9) | 27 (23.7) | 44 (18.4) |  |
| Histology |  |  |  |  |  |
| Clear cell | 196 (86.0) | 80 (89.9) | 101 (89.4) | 196 (83.1) | 0.272 |
| Non-clear cell | 32 (14.0) | 9 (10.1) | 12 (10.6) | 40 (16.9) |  |
| pT stage |  |  |  |  |  |
| pT1 – 2 | 181 (79.4) | 62 (69.7) | 89 (78.1) | 188 (78.7) | 0.282 |
| pT3 – 4 | 47 (20.6) | 27 (30.3) | 25 (21.9) | 51 (21.3) |  |
| Fuhrman grade |  |  |  |  |  |
| Low (Grade 1 – 2) | 66 (29.2) | 26 (29.9) | 36 (32.4) | 70 (29.8) | 0.943 |
| High (Grade 3 – 4) | 160 (70.8) | 61 (70.1) | 75 (67.6) | 165 (70.2) |  |
